# Endovascular Treatment of Thoracic Aortic Floating Thrombus in Patients Presenting with Acute Lower Limb Ischemia

**DOI:** 10.1155/2011/604362

**Published:** 2011-01-24

**Authors:** Nano Giovanni, Mazzaccaro Daniela, Malacrida Giovanni, Occhiuto Maria Teresa, Stegher Silvia, Foresti Davide, Tealdi Domenico Giuseppe

**Affiliations:** ^1^Division of Vascular Surgery, University of Milan and San Donato Polyclinic IRCCS, 20097 Milan, Italy; ^2^Postgraduate School of Vascular Surgery, University of Milan, 20122 Milan, Italy; ^3^Division of Vascular Surgery, San Donato Milanese, San Donato Polyclinic IRCCS, 20097 Milan, Italy

## Abstract

We report two cases of descending thoracic aorta floating thrombus treated with Bolton Relay thoracic free-flow stent graft. The patients had symptoms of lower limb ischemia; they underwent preoperative angiography and CTscan, then we proceeded with endovascular exclusion of the thrombus from the systemic circulation. At 12 months, the graft was still patent in both patients, without any signs of endoleak.

## 1. Introduction

Thoracic aortic mobile mural thrombus (TAMT) is a rare pathology and a potential source of cerebral, visceral, and peripheral embolism [1]. As for its rare occurrence, the literature shows no consensus about its aetiology or its appropriate therapy [2]. 

We report our experience in endovascular treatment of aortic thrombi in two cases using endovascular stent graft.

## 2. Case Reports

**Figure 1:**
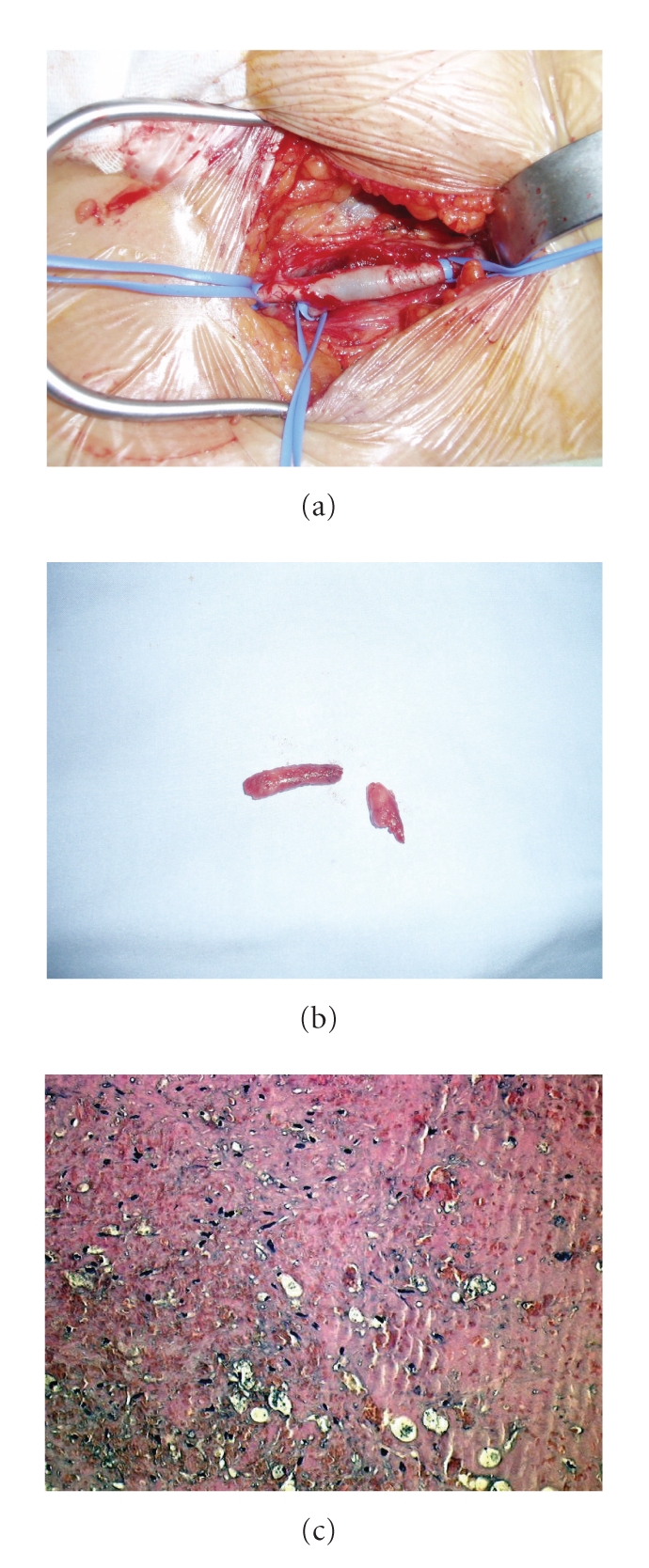
Intraoperative (a and b) and histological (c) findings.

A 45-year-old woman was referred to our hospital for an acute bilateral lower limbs ischemia. Clinical findings showed ischemic appearance of her distal legs and absence of peripheral pulses. She underwent a color-duplex ultrasound examination which documented thrombosis of both common femoral arteries, without any signs of atherosclerotic disease, so we performed an urgent embolectomy. The embolus macroscopically appeared as a fleshy smooth clot mixed with fresh portions of blood. Histological examination described it as fibrinous clot (Figure 1) while immunohistochemical and tumoral markers' searches were negative. 

Symptoms completely disappeared, but the source of embolism had to be identified. The patient was not a smoker and had no history of diabetes nor hypertension. She did not take any hormone-based medication, and electrocardiogram, chest radiography, and coagulation studies were normal. 

**Figure 2:**
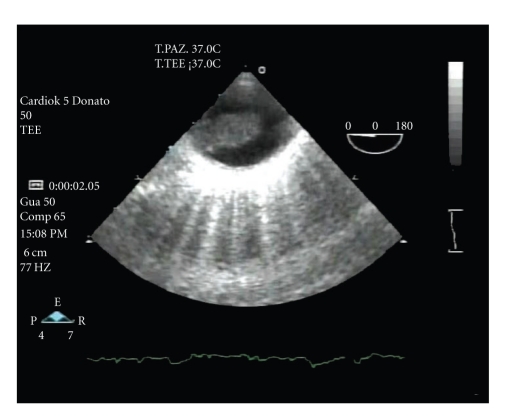
Transesophageal echocardiography documenting the floating mass in descending thoracic aorta.

Patient's medical history was negative for cardiovascular diseases, and a transesophageal echocardiography excluded a cardiac source of embolism whereas it detected an approximately 7.5 × 2.5 × 1.5 cm intraluminal polypoid floating mass in the proximal descending thoracic aorta (Figure 2). 

Ultrasound patterns of the mass were compatible with a diagnosis of thrombus, while intimal flaps, tears, or dissection were excluded. An angio-CT scan confirmed a free-floating lesion held by a pedicle from the descending aortic wall close to the isthmus. 

A contrast-enhanced MRI was then performed to exclude an aortic neoplasm. Collaterally, a huge uterine fibroma was discovered; anticoagulation was gained by heparin infusion and then followed by LMWH administration, and the patient underwent preliminary intervention of hysterectomy, in order to remove the mass which could obstruct our access to abdominal aorta and iliac arteries.

Treatment of thoracic thrombus was indicated to prevent any further embolic acute events, while its aetiology was still uncertain. Thoracic aorta's anatomy was favourable for endovascular coverage of the thrombosis site with a free-flow stent graft, without interfering with supra-aortic trunks vasculature so, four weeks later, the patient underwent endovascular coverage of the thrombus with a Bolton Relay thoracic free-flow stent graft under general anaesthesia. 

After a careful preoperative evaluation of thoracic aorta and femoral arteries diameters, we chose an aorto-aortic access below the renal arteries for endograft deliverage throughout a miniabdominal incisionFrom the left brachial artery a 5-Fr pigtail flush catheter was used to obtain an intraoperative aortogram which showed the extension of the thrombus. Aortography and trans-oesophageal echocardiography monitored endograft's correct delivery. 

A 22 mm × 10 cm Bolton Relay free-flow stent-graft was released in a portion of the aortic arch and descending aorta, covering the entire thrombus and its implantation site, to avoid embolism due to squeezing by radial-force and oversizing. 

**Figure 3:**
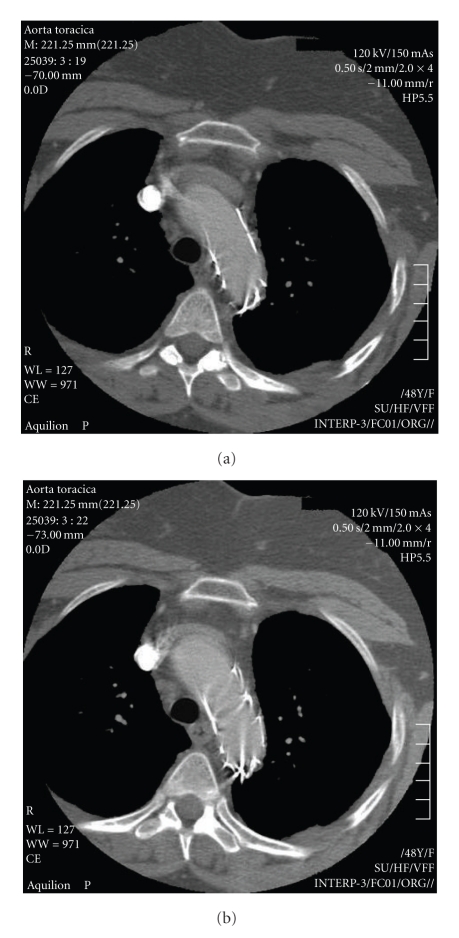
Angio-CT scan showing follow-up one year after intervention.

Postprocedural intrahospital period was free from any major complications. The patient was discharged 7 days later on oral lifelong anticoagulation, with an internationalized ratio between 2 and 3 to prevent any further emboli. At a followup of 1, 6, 12, 24, and 36 months Angio-CT scans showed patency of the graft no signs of endoleak (Figure 3); at clinical examination, there were no signs of neurological deficits nor signs of limbs ischemia.

A 67-year-old man arrived at our department, with recurrent feet pain and prostration.

The patient underwent clinical evaluation and laboratory tests which revealed a moderate renal failure with a creatinine blood level of 1.93 mg/dL. He was a strong smoker (more than 30 cigarettes/day) and had a history of medically well-controlled hypertension. He had dyslipidemia but, he had no previous history of diabetes; he had a history of stable angina, but, at the moment he was asymptomatic and electrocardiogram and chest radiography were normal. A color-duplex ultrasound evaluation of lower limbs was negative for obstructions and stenosis of major vessels.

**Figure 4:**
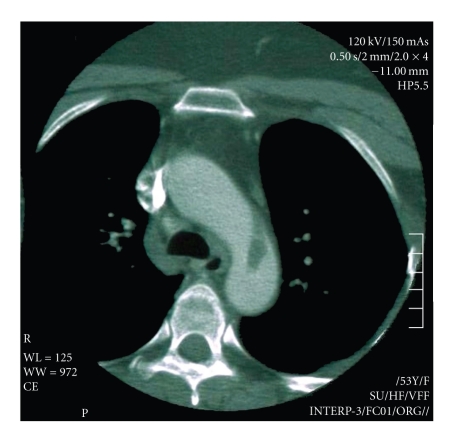
Angio-CT documenting the floating thrombus.

We then performed an Angio-CT scan which excluded intimal flaps, tears, or dissection, but revealed a floating mass attached by a small pedicle to the anterior wall of the proximal thoracic aorta (Figure 4). A contrast-enhanced MRI excluded an aortic neoplasm and suggested that it could be a possible thrombus. An endovascular treatment was indicated to exclude the embolic source, so anticoagulation therapy was administered to the patient (6000 IU of LMWH every 12 hours), with a moderate hydratation and Acetyl-cysteine the day before and the day after the procedure to prevent any renal damage due to contrast medium.

A Bolton Relay thoracic free-flow stent graft of 34 mm–100 mm was implanted under general anaesthesia, throughout a right femoral access for endograft delivery. 

A 5-Fr pigtail flush catheter was used to obtain an intraoperative aortogram which permitted us a safe delivery and excluded any involvement of the supra-aortic trunk. Once the femoral artery was isolated, we used a Lunderquist Extra Stiff (TS©MG-/-LES) 035/260CM guidewire; the device was placed to cover the entire thrombus and its implantation site. Aortography and trans-oesophageal echocardiography monitored endograft's correct delivery during the procedure.

During his length of stay, the patient's immunological and coagulation studies were normal and a total body CT-scan excluded any signs of neoplasms.

Procedure and postoperative in-hospital period were free from any major complications, and creatinine blood level was 0.87 mg/dL at discharge. The patient left the hospital 7 days after intervention with an oral lifelong anticoagulation with an internationalized ratio between 2 and 3, to prevent any further emboli.

**Figure 5:**
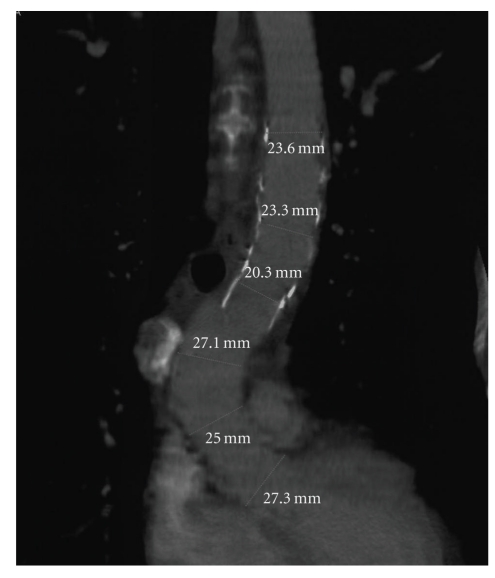
Angio-CT scan two years after endovascular repair.

During a 2-year follow-up, the patient was still asymptomatic. Serial angio-CT scans performed at 1, 6, and 12 months and thereafter annually after the intervention documented progressive shrinkage of thrombus between the aortic inner-lay and the endograft no signs of endoleak. In Figure 5 a multiplanar curve reconstruction using a Siemens workstation shows correct apposition of the graft 24 months after the intervention.

## 3. Discussion

Arterial embolism in approximately 80% of cases is a result of heart diseases, but also ulcerated atherosclerotic plaques in the aorta and carotid arteries are important noncardiac sources of emboli. Thrombosis and secondary embolism can also be associated with cancer, pregnancy, and hypercoagulation states, and in 10% of patients the source of peripheral embolism cannot be identified [3]. 

The first issue was about the identification of the source of peripheral artery embolism in a young patient without any signs of atherosclerotic disease nor coagulation diseases. In our first case, in fact, we performed clinical examination, laboratory findings, electrocardiography and finally-angio-CT scan to exclude rare forms such as foreign- body embolism, septic embolism, embolism from malignant tissues, embolization from cardiac myxoma or from a myxomatous atrial septal defect [3]. 

The second issue concerned the diagnosis of the mass' aetiology. We suspected an angiosarcoma, a rare disease [4] which generally grows up from endothelium and develops intraluminally as a polypoid mass narrowing aortic lumen [5]; it represents a potential source of emboli and can cause thrombosis in small vessels after intimal invasion. Its diagnosis is difficult because it must be differentiated from atherosclerosis, dissection or acute aortic syndrome [5], and thrombus [6]. Angio-CT scan and contrast-enhanced-MRI are the most sensitive diagnostic methods [5], but also aortic biopsy with radial-jaw forceps through femoral access and IVUS-guidance has been described [7]. Moreover, histology and immunohistochemical markers are useful to confirm the nature of the mass [6–8]. We excluded also a penetrating aortic ulcer (PAU), which is usually asymptomatic, confined to the intimal layer, and is associated with a localized dissection. 

In our first case, the nature of the mass was confirmed by a histological sample, while in the second case CT-scan and MRI preoperative imaging excluded any other kind of lesions.

We suppose the thrombus in the second case had probably been caused by atherosclerotic disease due to smoking history, while in the first case we are still uncertain about its aetiology; the only finding was a huge uterine fibroids, which, however, may not be related to the aortic thrombus. 

The third issue concerned the choice of the appropriate therapy. Traditional open repair is justified in well-qualified units, better when there are no signs of systemic embolism [1]. Surgical approach includes local thrombectomy or endarterectomy, aortic segmental resection and prosthesis substitution [9]. Anticoagulation has been described, too, in case of small uncomplicated thrombi from coagulation disorders [10]. Conservative management with only systemic anticoagulation would however carry significant risks of embolization, as for the nature of the mass; moreover, there is no strong evidence as to the duration or success of this strategy.

In the last decade materials and techniques for endoluminal treatment of these thoracic pathologies have improved [11] and perioperative complication rates seems to be lower than open approach, even if embolic events during endovascular procedure are well described [12]. Midterm results of 24-month followup are encouraging, while long-term results are still to be discussed. In our first case, we preferred a less invasive approach rather than an open approach with extracorporeal circulation, while in the second case endovascular coverage was chosen for patient's comorbidities. As for the choice of the graft, we preferred the Bolton Relay because we are used to this type of endograft. Moreover, the presence of a free-flow permitted a good proximal landing zone without covering the left subclavian artery.

Finally mobile thrombus of the thoracic aorta is a rare pathology which needs to be diagnosed as soon as possible for serious complications that can occur mostly for embolism. As for its aetiology, differential diagnosis should be done with angiosarcoma, aortic dissection, and PAU.

About its treatment, endovascular exclusion of the floating thrombus is a feasible and less invasive alternative to open surgery.
